# Examining Poverty Reduction of Poverty-Stricken Farmer Households under Different Development Goals: A Multiobjective Spatio-Temporal Evolution Analysis Method

**DOI:** 10.3390/ijerph191912686

**Published:** 2022-10-04

**Authors:** Yanhui Wang, Shoujie Jia, Wenping Qi, Chong Huang

**Affiliations:** 1 Key Laboratory of 3-Dimensional Information Acquisition and Application, Ministry of Education, Capital Normal University, Beijing 100048, China; 2 China Siwei Surveying and Mapping Technology Co., Ltd., Beijing 100086, China; 3 Institute of Geographic Sciences and Natural Resources Research, Chinese Academy of Sciences, Beijing 100101, China

**Keywords:** multiobjective, poverty reduction and development, spatio-temporal evolution, poverty factors

## Abstract

Accurately identifying the degree of poverty and poverty-causing factors of poverty-stricken farmer households is the first key step to alleviating absolute and relative poverty. This paper introduces a multiobjective spatio-temporal evolution analysis method to examine poverty reduction of poverty-stricken farmer households under different development goals. A G-TOPSIS model was constructed to evaluate poverty-stricken households under short-, medium-, and long-term development goals. Then, GIS analysis methods were employed to reveal the spatio-temporal distribution of poverty-stricken households, and poverty causing factors were detected using the obstacle degree model. Taking Fugong County in Yunnan Province, China, as an example, the empirical results show that: (1) Great progress has been made in poverty reduction during the study period; however, some farmer households which have escaped absolute poverty are still in relative poverty and are still highly vulnerable. (2) Farmers with higher achievement rates under three different development goals are mainly distributed in the central and northern regions of study area, with a pattern of high–high agglomeration under the medium and low development goals, while low–low agglomeration mostly appears in central-southern regions. (3) Under the short-term development goals, the main poverty-causing factors are per capita net income, safe housing, sanitary toilets, years of education of labor force and family health. Under the medium- and long-term goals, per capita net income, labor force education and safe housing are the development limitations. (4) Infrastructure and public service are crucial to ending absolute poverty, and the endogenous force of regional development should be applied to alleviate the relative poverty through sustainable development industries and high-quality national education.

## 1. Introduction

Poverty is a common problem faced by governments around the world. It can effectively hinder the sustainable development of mankind and the harmonious continuation of civilization. It is also one of root causes of social conflicts. The gap between rich and the poor is becoming more and more pronounced in today’s epidemic-distressed world, and alleviating poverty is becoming a key goal for societies which are striving for progress and development. Therefore, the Sustainable Development Goals (SDGs) set forth by United Nations are intended to express the global aspiration of “shared blueprint for peace and prosperity for people and the planet, now and into the future’’. As the first goal of SDGs, “eliminate all forms of poverty in the world’’ intends to eliminate poverty around the world, which, undoubtedly, means that the reduction of extreme poverty is the basis of sustainable development.

Globally, in 2020, 1.3 billion people were still living in multidimensional poverty [1]. China is no exception, especially when it comes to farmer households in rural areas. In 2012, the number of people living in poverty in China was 98.99 million. Since 2013, China has carried out the policy of ‘targeted poverty alleviation programs’ to reduce overall absolute poverty, and significant achievements have been made. By the end of 2020, a total of 832 state-level poverty counties had been lifted out of absolute poverty. However, the elimination of absolute poverty under the current standard does not mean the end of poverty. Disasters, epidemics, unsustainable alleviation of temporary poverty and relative poverty are all powerful hindrances for high-quality economic development and rural revitalization. Anti-poverty strategies should therefore shift their focus from poverty alleviation to the establishment of a long-term mechanism to solve relative poverty, i.e., to achieve high-quality social sustainable development. 

While the degree of absolute poverty can be accurately described by several simple indicators, accurately evaluating the degree of the relative poverty for poverty-stricken farmers remains challenging. The process involves many complex factors at both the individual and community levels. Moreover, these factors are interconnected and evolving under different developing goals. The traditional single-objective static assessment model cannot meet the requirements. Therefore, we hypothesize that a multiobjective spatio-temporal evolutionary measurement model—which fully considers the unbalanced development of both farmer households from the micro level and multidimensional developments from the macro level—may have the potential to accurately evaluate poverty reduction effectiveness and identify the spatio-temporal evolution pattern of relative poverty. Focusing on these problems, this paper carries out methodological research and an empirical analysis.

## 2. Literature Review

From the perspective of a literature review, Amartya Sen (1981) [2] put forward the multidimensional poverty theory based on the perspective of “feasible ability”, which deepens our understanding of poverty on subjective and objective levels and expands the one-dimensional vision of welfare economics to the multidimensional field. With the concept of poverty expanding from one dimension to multidimension, it is now common to grasp and measure the essence of poverty from a multidimensional perspective [3,4]. Multidimensional poverty measurement methods are also emerging. Sen’s theory of feasible ability method defines and measures poverty from the perspective of human development. In 2010, the United Nations Development Programme (UNDP) published the multidimensional poverty index (MPI) based on the A–F method proposed by Alkire and Foster in 2007 [5]. This expanded the measurement of poverty in human development theory. Meanwhile, scholars have also successively adopted a variety of methods for the identification, measurement and analysis of multidimensional poverty, including the Gini coefficient [6], FGT index [7], ELES model [8], A-F double critical value method [4,5], fuzzy evaluation [9], comprehensive index method [3], TOPSIS (Technique for Order Preference by Similarity to an Ideal Solution) [10], and so on, for proposal explanations or empirical application. These studies show that the trend of evaluating poverty using multidimension comprehensive poverty metrics rather than single dimension economic ones has become mainstream. For example, the TOPSIS model showed a good effect in multiobjective decision-making analyses by evaluating the degree of similarity between the research object and the idealized value [11,12]. For its application in poverty identification, the traditional TOPSIS model had been used to measure the degree of relative poverty and internal differences among impoverished groups [13,14]; however, it only uses Euclidean distance to express the comprehensive closeness of the object of interest to the development goal, and it is easy to cover up real poverty representation information of other indicators because of the large deviation of one indicator from the target value. This could lead to a deviation in the targeting and measurement of poverty, making this approach unable to objectively reflect the deprivation of each index of the object to be evaluated. 

As far as the poverty line is concerned, most of the aforementioned studies only took the absolute poverty line as the reference, so it is difficult to accurately identify the relative poverty gap and its causes under different poverty reduction and development goals. Su and Zhao [15] stated that relative poverty means that individuals or families are in a state where they could meet the basic survival needs but have not reached the average living standard of their society, manifesting in inequality of education, medical care, living standards and other dimensions. Therefore, the measurement of relative poverty should also turn to multidimensional and multiobjective approaches [16]. However, current research is mainly focused on the multidimensional identification and evaluation of poverty reduction under a single goal. As such, there is a lack of connection between relative and absolute poverty, and between long- and short-term goals. 

With the improvement of GIS and RS technology, the spatial distribution pattern of poverty has become a research hotspot. For example, Xia et al. [8] and Jiang et al. [17] used spatial autocorrelation analysis methods to make an empirical analysis of the spatial distribution and agglomeration characteristics of poverty. Wang and Chen [18] and Ge et al. [19] used Ripley’s K function and other GIS spatial analysis methods to examine the spatial agglomeration distribution of poverty-stricken households. However, there were some deficiencies in the application of spatio-temporal statistical analysis methods in these studies. For example, the methods applied to spatial cross-section data, such as Moran’s I index, nuclear density statistics, Getis-Ord G* statistics and other traditional methods, are mainly used for comparative descriptive analyses on a time axis. Therefore, it is necessary to introduce spatio-temporal statistical methods into spatial analyses of poverty and to identify correlations among the results of cross-sectional spatial statistical analyses over time in order to grasp the effectiveness and dynamic development trends of poverty reduction and development. Dai et al. [20] and Xu et al. [21] analyzed the spatio-temporal evolution characteristics of road traffic, rainfall and snow disasters based on Moran’s spatio-temporal I index. Xia et al. [22] analyzed the temporal and spatial dynamic characteristics of poverty in ecologically fragile Karst areas based on the Lisa time path and Lisa temporal transition methods. These spatio-temporal statistical models can quantitatively reveal the spatial-temporal distribution patterns of poverty, but their application in the field of spatial analysis of poverty is obviously insufficient.

To sum up, although a consensus that poverty is multidimensional, dynamic, complex, and regional is slowly forming, most studies continue to focus on the identification and evaluation of poverty according to a single goal. There is still a lack of convergence between relative poverty measurement and absolute poverty identification, and between long- and short-term goals. Meanwhile, to some extent, it remains difficult to adapt existing models to identify and dynamically monitor relative poverty under multiple goals such as the United Nations sustainable development goals, rural revitalization goals or targeted poverty alleviation goals. Additionally, they cannot directly reflect the poverty level and development gap of poor farmers under multiple objectives. Spatio-temporal analyses are mainly based on the spatial econometric model which lacks the application cases of the spatio-temporal econometric model in poverty research, resulting in difficulties in accurately investigating the spatio-temporal development characteristics of multidimensional impoverished farmers.

In this context, this paper uses Fugong County in China as an example, aiming at poverty reduction and development monitoring of poor farmers under different development goals. It proposes a G-TOPSIS comprehensive evaluation model to identify poverty degree and type under three different goals, and reveals the spatio-temporal evolution and poverty types of multidimensional poverty-stricken farmers by introducing spatio-temporal econometric methods and an obstacle degree model. It is expected to provide an effective technical decision-making basis for the design of forward-looking policies for China and other developing regions to alleviate relative poverty, as well as for the implementation of national rural revitalization and sustainable development strategies.

## 3. Study Area and Data Processing

### 3.1. Study Area

**Figure 1:**
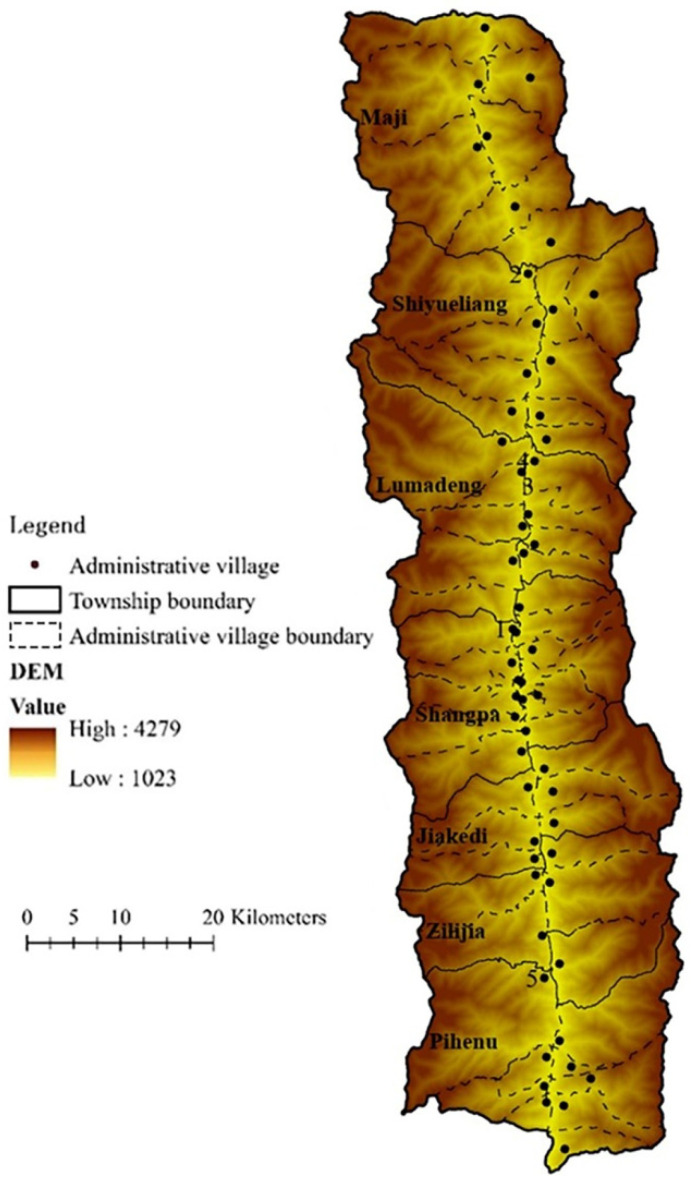
Overview of study area. (Administrative Village No.: 1. Shidi, 2. Lamadi, 3. Watuwa, 4. Chisadi, 5. Wawa).

We selected the Fugong county of Yunnan province in China as the study area. As shown in Figure 1, Fugong county is in the Nujiang gorge between Biluo and Gaoligong Mountains, in the middle of the Hengduan Mountains in Northwest Yunnan. It has seven Towns and a total of 58 administrative villages. From north to south, the seven towns are Maji, Shiyueliang, Lumadeng, Shangpa, Jiakedi, Zilijia and Pihenu. 

The population of Fugong County is distributed in a line along the Nujiang Valley. As a key deep-poverty county of national contiguous poverty-stricken areas of China, Fugong is also largely inhabited by minority ethnic groups, including the Lisu, Nu, Bai, Naxi and 20 others. Meanwhile, Fugong is also a frontier county between China and Myanmar and is mountainous, with an altitude of 1023~4279 m. The county faces a lot of problems such as traffic congestion, a lack of infrastructure and frequent occurrences of natural disasters like landslides and mudslides, which directly lead to the low-level and unsustainable economic development of the area. 

### 3.2. Data Source and Preprocessing

The data used in this study include socio-economic and geographic data. The socio-economic data mainly came from a survey data on poor farmers in 2015–2018, issued by the local poverty alleviation department of Fugong county, as well as the statistical yearbook of China and Yunnan Province, China Poverty Alleviation and Development Statistical Yearbook in the same period. Geographic data included digital elevation model (DEM) data with 30 m resolution, boundary vector data of administrative villages and towns and other geographic data from the Geospatial Data Cloud (http://www.gscloud.cn, accessed on 5 March 2020). All the above datasets were preprocessed by georeferencing, splicing, clipping, etc.

## 4. Methods

The study period of 2015–2018 was a critical period of poverty alleviation in China. Poverty-stricken households were not only implicated in the short-term goal of eliminating absolute poverty, but also the long-term goal of alleviating relative poverty and achieving sustainable poverty alleviation. Therefore, according to the strategy of getting rid of China’s customized rural absolute poverty line, i.e., achieving the local average development level in Yunnan and the average development level in China, this research designed a multiobjective development evaluation model called G-TOPSIS to identify the poverty and development levels of farmers under three different development goals, i.e., a short-term development goal (reaching the national standard of eliminating absolute poverty), a medium-term development goal (reaching the provincial average development level of rural residents of Yunan province) and a long-term development goal (reaching the national average development level of rural residents of China). Meanwhile, a spatio-temporal statistical methods, e.g., spatio-temporal autocorrelation analysis, is introduced to analyze the spatio-temporal distribution pattern of multidimensional development among poor farmers. Finally, an obstacle degree model is proposed to identify the types of poverty experienced by farmers under different goals.

### 4.1. Multiobjective Poverty Reduction Measurement Model 

#### 4.1.1. Multiobjective Poverty Reduction Measurement Indicator System

Aiming at the problems of absolute poverty, relative poverty and unbalanced development that poverty-stricken households are facing, and considering the poverty reduction and development needs of the national targeted poverty alleviation strategy, the rural revitalization strategy and the sustainable development strategy of China, a multiobjective poverty reduction measurement indicator system for poverty-stricken households is needed. 

As stated, a reasonable multidimensional poverty framework for a multiobjective poverty measurement indicator system should include three key types of elements, reflecting livelihood, vulnerability and social exclusion perspectives of multidimensional poverty [4,16,17]. The multiobjective poverty measurement indicator system should not be an exception, in that the framework must serve effectively to analyze internal interactions among structural and individual factors that lead to absolute and relative poverty. In the system framework, stable income and living conditions are the intrinsic bases for sustainable livelihoods, and the ability of livelihood capital conversion is the ability to respond to livelihood risks such as diseases, unexpected natural disasters, and other vulnerability factors. On the other hand, the path dependence of livelihood capital conversion maintains social stratification and leads to social exclusion. Additionally, the fairness and justice of social mechanisms such as education and medical care are of great significance for reducing social exclusion or promoting social integration.

At the specific indicator level, we strived to ensure the rationality and typicality of indicators, the feasibility of obtaining indicator data, the relevance to and operability with China’s poverty alleviation criteria, and the actual situation of regional development in the study area [10,16,18]. In this respect, we established a candidate indicator system including six dimensions, i.e., housing safety, living conditions, education, health, family income, and social security. Then, the candidate indicators were screened using the coefficient of variation method, resulting in a system with six dimensions and 10 basic indicators; see Table 1. 

#### 4.1.2. Development Goals and Relative Poverty Lines

In recent years, China’s general rural development policies have focused on consolidating the effect of the alleviation of absolute poverty, achieving the development goals of Rural Revitalization, and promoting the prosperity of the people as a whole. However, different regions are in different stages of development and have different needs in terms of poverty reduction and sustainable development. Therefore, the average development level of rural residents in China and Yunnan province were respectively selected as the reference objects in the study area. In other words, in order to reveal inequalities in income, living standards, and resource distribution among poor households, we followed the relative nature of poverty and the actual development situation of farmers in the study area. We set the threshold as the elimination of absolute poverty, as outlined by China’s precise poverty alleviation strategy, as the reference short-term goal (T_1_). We used the average development level of rural areas in the whole province of Yunan as the reference for the medium-term goal (T_2_), and the average development level of rural residents in the whole nation as the reference for the long-term development goal (T_3_). 

In our specific benchmarking of a relative poverty line for each indicator under the three aforementioned goals, the value of popularizing public services and infrastructure construction refer to the relevant national development planning standards of China, according to the characteristics of regional development and the overall requirements of ensuring the improvement of people’s sense of well-being and happiness. On the other hand, the indicators of education and income, which are difficult to achieve, were assigned with reference to the national poverty alleviation standards of China and the provincial average levels of Yunnan and the whole country of China, respectively.

#### 4.1.3. Multiobjective Poverty Measurement Method with G-TOPSIS

Poverty is a phenomenon which is reflected in multidimensional indicators of farmer households. Therefore, the Technique for Order Preference by Similarity to Ideal Solution (TOPSIS), proposed by Hwang and Yoon in 1981, also known as the advantage and advantage solution distance method, could be used to compare and rank poverty-stricken households by calculating their closeness to an ideal state [13,23]. 

TOPSIS is a commonly used intragroup comprehensive evaluation method. It can make full use of the information contained in the original data, and its results can accurately reflect the gap between the evaluation indexes, i.e., to judge the advantages and disadvantages of each index in the data according to an existing data. Its core principle is to first determine the optimal ideal value (positive ideal value) and the worst ideal value (negative ideal solution) of each index. The former is the best value of an assumption; its attribute values reach the best value of each candidate index value. Meanwhile, the negative ideal solution is the worst value of another assumption. The weighted Euclidean distance between each actual index value and positive and negative ideal value is calculated to obtain the proximity between each actual index value and the most ideal value. The smaller the distance to the optimal solution, the better.

However, this traditional TOPSIS model uses the Euclidean distance to express the closeness of the object of interest to the development goal. In this way, it is easy to cover up real poverty identification information of other indicators because of large deviations of some indicators from the target value, leading to a deviation in the targeting and measurement of poverty. In order to overcome the index measurement deviation caused by using only Euclidean distance, this paper introduces a goal completion coefficient (called *G* coefficient), building a G-TOPSIS model to objectively reflect the deprivation of each index of the object to be evaluated. The G-TOPSIS model calculates the comprehensive closeness degree of each poor farmer household relative to different development goals (i.e., *T*_1_, *T*_2_ and *T*_3_ in this paper), and then summarizes the goal achievement rates, i.e., *H*_1_ (short-term), *H*_2_ (medium-term) and *H*_3_ (long-term), corresponding to *T*_1_, *T*_2_, and *T*_3_, respectively. The implementation of G-TOPSIS model includes the following three steps.

(1) Data standardization and index weighting

The extremum standardization method was used in this study to standardize the measurement index data and each development goal, and the dimension unit of each index was removed according to the positive and negative directions of that index; therefore, the larger the standardized value, the better the development status. Household income and its relative indicators were revised on the basis of the national constant prices of 2011. The analytic hierarchy process (AHP) and entropy value method were used to determine the weights of each index subjectively and objectively, and then the optimized combination weight of these subjective and objective weights was calculated based on game theory [4,18]. The calculation process is as follows:

Firstly, the weight vectors of both the AHP and entropy value methods are shown in Formulas (1) and (2):

(1) $$\omega =\left(\omega_{1},\omega_{2},\cdots ,\omega_{m}\right)\;$$

(2) $$u=\left(u_{1},u_{2},\cdots ,u_{m}\right)$$

Then, the optimized weight value matrix based on game theory was calculated as follows:

(3) $$\left(\begin{array}{cc} \omega \cdot \omega^{T} & \omega \cdot u^{T}\\ u\cdot \omega^{T} & u\cdot u^{T} \end{array}\right)\left(\begin{array}{c} \alpha_{\omega }\\ \alpha_{u} \end{array}\right)=\left(\begin{array}{c} \omega \cdot \omega^{T}\\ u\cdot u^{T} \end{array}\right)$$

where $\alpha_{\omega }$ and $\alpha_{u}$ denote the weight values of the AHP and entropy value methods, respectively, and the combined weight, i.e., *w*, is depicted as follows:

(4) $$w=\alpha_{\omega }\cdot \omega +\alpha_{u}\cdot u$$

where each dimension and each indicator within the corresponding dimension should be subject to the following conditions:

(5) $$\omega_{1}+\omega_{2}+\cdots +\omega_{n}=1$$

(6) $$\omega_{i1}+\omega_{i2}+\cdots +\omega_{im}=1$$

where $\omega_{i}$ indicates the weight value of each dimension *i* and $\omega_{ij}$ is the weight value of each indicator *j* in dimension *i*. 

According to the above process, the combined weight of each indicator and dimension is shown in Table 1.

(2) Calculating the closeness degree

The first step is to calculate G coefficient, which considers the difference of the impact of various indicators on poverty-stricken households and reflects their deprivation degree in different dimensions. The higher the G value, the more indicators are required to achieve the development goals and the closer they are to the development goals. For each farmer household in different situations, with reference to the development goals *T* {*T*_1_, *T*_2_, …, *Tj*}, we judged whether the development indicators of poor farmers reached the goals. Then, the completion coefficient *G* of the poverty measurement indicators was obtained with the following formula.

(7) $$G=\sum_{i=1}^{n}w_{j}g_{j}$$

where *w*_j_ is the weight of indicator *j*. If the *j* index of the farmer household reaches the target goal, *g_j_* is set at 1; otherwise, *g_j_* is set to 0. When G is 1, it means that all dimensions of multidimensional poor individuals have achieved the development goals. 

Secondly, for each farmer household *i*, the European distance index *L*_i_ of the development situation from certain ideal goal was calculated. The distance index *L*_i_ represents the degree to which the evaluation indicator is close to the ideal value, including the distance from the evaluation vector to the positive and negative ideal solutions, *D*+ and *D*^−^, respectively. Its calculation formula is as follows:

(8) $$L_{i}=\frac{D^{-}}{D^{+}+D^{-}}=\frac{\;\sqrt{\sum_{j=1}^{m}w_{j}{\left(x_{ij}-v_{j}{}^{-}\right)}^{2}}}{\;\sqrt{\sum_{j=1}^{m}w_{j}{\left(x_{ij}-v_{j}{}^{+}\right)}^{2}}\;+\sqrt{\sum_{j=1}^{m}w_{j}{\left(x_{ij}-v_{j}{}^{-}\right)}^{2}}}$$

where *X*_ij_ represents the normalized value of indicator *j* of poor household *i*, *W*_j_ represents the weight of indicator *j*, *V*_j_ + represents the normalized maximum value (the ideal goal value), and *V*_j_—denotes the normalized minimum value (the least ideal value).

Finally, the comprehensive closeness degree *Y*_i_ was calculated. For each farmer household *i*, the comprehensive closeness degree *Y*_i_ can reflect the closeness degree of the development level of *i* to the ideal goal, and the ranking result of *Y*_i_ can be regarded as the household development level. The lower the *Y*_i_ value, the more serious the deprivation of the household, i.e., the greater the relative gap with the ideal state, and the lower the development level. *Y*_i_ may be calculated as follows:

(9) $$Y_{i}=G_{i}L_{i}$$

where *G_i_* represents the completion coefficient of *i*, and *L_i_* represents the distance index of *i*.

(3) Judging the realization rate of development goals

The ranking results of comprehensive closeness degree of poverty-stricken households corresponding to each development goal were used to determine whether the development goal had been achieved or not. Realization rate *H* of the development goal of poverty-stricken households in each region (i.e., county, town, or village in this study) was obtained based on a regional summary of all farmer households in that region, as follows.

*H = q/n* (10)

where *q* is the number of farmer households that have achieved the development goal in the region and N is the total number of farmer households in the region.

Similarly, the calculation method of the target realization rate *Hj* of each indicator *j* was as follows:

(11) Hj=qjn

where *H_j_* is the target realization rate of indicator *j*, *q_j_* is the number of farmer households in the region that have reached the development goal in indicator *j*, and *n* is the total number of farmer households in the region.

### 4.2. Spatio-Temporal Autocorrelation Analysis Model

Spatial autocorrelation is the correlation among values of a single variable across a two-dimensional surface that are locationally referenced or tied together by an underlying spatial structure, introducing a violation of the independent observations assumption of classical statistics [24]. Spatial autocorrelation refers to the potential interdependence between the observed data of some variables in the same distribution area, where the adjacent observations with similar data values show positive spatial autocorrelation on the map, and those observations with contrasting values tend to show negative spatial autocorrelation. It helps to illustrate the degree to which one object is similar to other nearby objects, and is usually taken to indicate that there is something of interest in the distribution of map values that calls for further investigation in order to understand the reasons behind the observed spatial variation [25]. It can be quantified with various indices, such as the Global Moran’s I, Local Moran’s I or the Getis-Ord Gi* [17,18]; however, these approaches were not applied to the spatio-temporal case (including many time steps) because of computational limitations [26]. Spatio-Temporal autocorrelation is helpful to be able to identify autocorrelation in space and time simultaneously. 

Based on the global and local spatial autocorrelation principles, this paper introduces the Spatio-Temporal Moran’s I, i.e., STI, to analyze the spatio-temporal structure of variables [27,28]. In this study, the development state of administrative village units in a certain year was defined as the Spatio-Temporal object, and the global STI index was constructed to analyze the spatial and temporal evolution law of multidimensional poor household development. If STI is greater than 0, it means that the development of multidimensional poverty-stricken households presents a global positive correlation and shows the characteristics of spatio-temporal aggregation. In contrast, it shows that the development of multidimensional poverty-stricken households is negatively correlated with the overall situation and reveals the characteristics of spatio-temporal dispersion. The calculation method is as follows:

(12) $$\mathrm{STI}=\frac{NT\sum_{p=0}^{N}\sum_{i=0}^{T}\sum_{q=0}^{N}\sum_{j=0}^{T}w_{\left(p,i\right)\left(q,j\right)}\left(y_{\left(p,i\right)}-\bar{y}\right)\left(y_{\left(q,j\right)}-\bar{y}\right)}{\sum_{p=0}^{N}\sum_{i=0}^{T}{\left(y_{\left(p,i\right)}-\bar{y}\right)}^{2}\times \sum_{p=0}^{N}\sum_{i=0}^{T}\sum_{q=0}^{N}\sum_{j=0}^{T}w_{\left(p,i\right)\left(q,j\right)}}$$

where *N* represents the number of spatial sequences of spatio-temporal objects (58 in this paper), T represents the number of time series (4 in this study), and *W (p,i)(q,j)* represents the space-time weight matrix. When two spatio-temporal objects have an adjacency relationship in both the space and time dimensions, the value is 1; otherwise, the value is 0. *y_(p,i)_* and *y_(q,j)_* represent the values of spatio-temporal objects *(p,i)* and *(q,j)*, respectively, and $\bar{y}$ represents the average value of all spatio-temporal objects. Due to the “long and narrow” geographical characteristics of the research area, “shared boundary or node” mode was selected to define the spatial relationship method.

### 4.3. Poverty-Causing Factors Detection Model

Analyzing the influencing factors of spatial poverty traps has important practical significance for local people to overcome poverty and improve their sustainable development abilities. Such analyses are also necessary to evaluate the obstacle effect of single and classified indexes to examine key constraints of different poverty-causing factors. 

Based on the multidimensional poverty development evaluation index system for poor households, the impact of each index on the poverty reduction and development of a poor household under different standards is calculated through the obstacle degree measurement model. In this way, the poverty causing factors under different development levels were analyzed. Referencing the principle of obstacle degree measurement model [29], the diagnosis of obstacle factors in our study introduced three indexes: deviation degree (*I*_j_), factor contribution degree (*w*_j_) and obstacle degree (*C_j_*), i.e., the weight of index *w*_j_ to represent the deviation contribution degree of index *j*, and *I*_j_ to represent the gap between index *j* and development goal, expressed as *I_j_ = t_j_*−*x_j_.* When the index *j* reaches the supposed development target *t_j_*, *I*_j_ is equal to 0. The greater the value of *C_j_*, the greater the influence of the index on individual poverty, allowing us to identify the main poverty-causing factors. The formula is as follows:

(13) $$C_{j}=\frac{\;w_{j}I_{j}}{\sum_{j=1}^{m}w_{j}I_{j}}$$

## 5. Results and analysis

### 5.1. Overall Multiobjective Development Status of Poor Farmer Households

**Figure 2:**
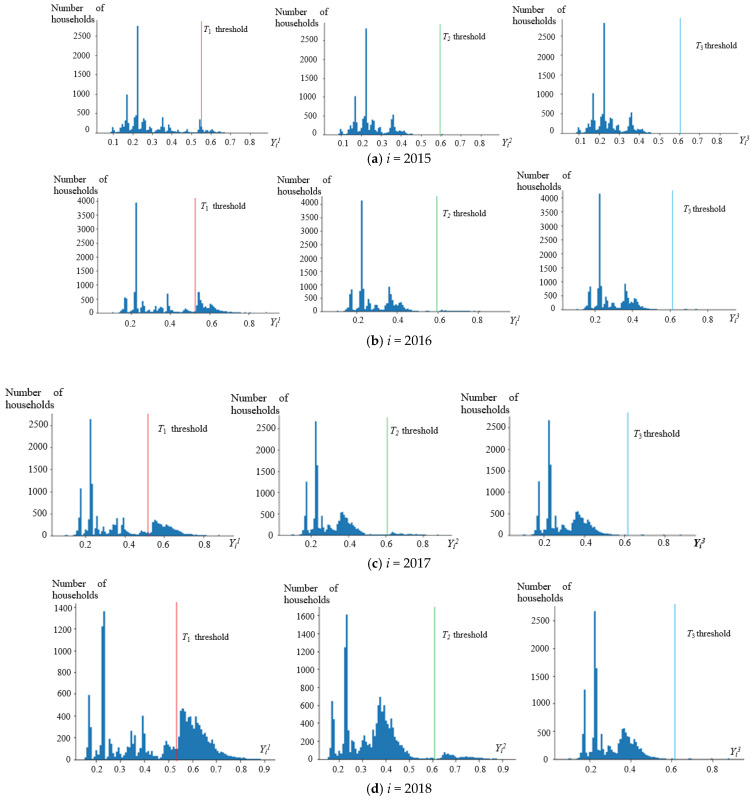
Statistical distribution of comprehensive closeness degree of poor households for *Y_i_^1^_,_* (**left**), *Y_i_^2^* (**middle**), and *Y_i_^3^* (**right**) in Fugong county from 2015 to 2018 (**a**–**d**).

Using the G-TOPSIS method to evaluate the multiobjective development level of poor households, the comprehensive closeness degree, *Y_i_^1^_,_ Y_i_^2^* and *Y_i_^3^* in year *i*(*i*∈{2015, 2016, 2017, 2018}), under the short- (*T*_1_), medium- (*T*_2_), and long-term(*T*_3_) goals of poor farmers in Fugong County from 2015 to 2018 were calculated; the statistical distribution is shown in Figure 2a–d, respectively. Under the short-term national poverty threshold standard of *T*_1_, comprehensive closeness degree *Y_i_^1^* was 0.53, 0.53, 0.54 and 0.55, respectively. The comprehensive closeness degree *Y_i_^2^*, which was used to evaluate the medium-term average development level of rural residents in Yunnan of *T*_2_ threshold. was 0.59, 0.60, 0.61, 0.61, respectively. Finally, the comprehensive closeness degree *Y_i_^3^* of the long-term average development level of rural residents in China of *T*_3_ threshold was 0.60, 0.61, 0.61, 0.62, respectively. By comparing these numbers in order, *H*_1_, *H*_2_ and *H*_3_, which represented the short-, medium-, and long-term the realization rates, were obtained. Then, the development levels of poor households were divided into the following four levels: low (under the national poverty threshold standards), fairly low (higher than the national poverty threshold standards but lower than the average level of Yunnan province), relatively high (higher than the average level of Yunnan province but lower than the national average level), and high (higher than the national average level). 

Figure 2 shows that under the short-term development goal of *T*_1_, from 2015 to 2018, the average comprehensive closeness degrees *Y_i_^1^* of Fugong county were 0.39, 0.44, 0.51 and 0.56. The comprehensive closeness degree increased year by year, and the distance between absolute poverty and ideal state was gradually reduced. By 2018, the average level of poor households in the county exceeded the nation’s poverty threshold standards; there were 7903 poor rural households which achieved the short-term development goal of moving out of absolute poverty, and the overall short-term goal the realization rate (H^1^) was 54.83%. In terms of the statistical distribution characteristics, the nation’s poverty threshold standard line for *T*_1_ divided the distribution of *Y_i_^1^* into two sections. There was no obvious statistical distribution trend on the left side of *T*_1_ threshold line, which means that those presented on this side have not reached the nation’s poverty threshold standards. The distribution near the *T*_1_ threshold indicates that the development of peasant households which have been lifted out of poverty is inadequate, and most of them remain near the absolute poverty line.

Under the mid-term development goals of *T*_2_, the calculated the average comprehensive closeness degree *Y_i_²* of Fugong County was 0.36, i.e., far below the value calculated (0.61) based on the average level of development among rural residents in Yunnan Province. By 2018, a total of 1042 poverty-stricken households were above the average development level of the province of Yunnan. Finally, from 2015 to 2018, the medium-term target achievement rate H² was 0.1%, 1.57%, 3.53% and 7.23%, respectively.

Under the long-term development goals of *T*_3_, the calculated average comprehensive closeness degree *Y_i_^3^* of Fugong was 0.35, i.e., much lower than that calculated based on the average level of development of rural residents for the whole country (0.62), By 2018, there were 35 poverty-stricken households above the national average development level. However, the achievement rate of long-term development goals H³ was only 0.24%, which indicates that the overall proportion was low. Compared with the average level of the whole province and the whole country, the development level of poverty-stricken households in Fugong County still has a long distance to go, indicating that poverty rate of Fugong County is relatively high. 

The development levels of poverty-stricken households are divided according to the realization ratio of the development goals. As shown in Table 2, from 2015 to 2018, poverty-stricken households with low development levels accounted for 92%, 74.8%, 68.93%, 45.17%, respectively. Additionally, the proportion of poverty-stricken households with relatively low development levels was 6.99%, 23.6%, 27.54%, 47.60% respectively. There were 6511 households in the low development level, accounting for 45.17% of the total. Furthermore, the number of poverty-stricken households in the relatively low level of development was 6861, i.e., 47.60% of the total. Meanwhile, there were 1007 poverty-stricken households with relatively high level of development status, making up 6.99% of the total. The number of poverty-stricken households with a high level of development was 35, accounting for 0.24% of the total. It can be therefore seen that the development levels among poverty-stricken households in Fugong County mainly evolved the low to relatively low, and the total distribution proportion of the low and the relatively low levels of development were both higher than 90%. Therefore, although poverty-stricken households have made great progress, the overall development levels among poverty-stricken households are still at a relatively low level. In 2018, there were still a large number of poverty-stricken households in Fugong County, which means that a great deal of work remains to be done. In summary, eliminating absolute poverty is the most urgent task for Fugong County. Although some poverty-stricken households have already escaped absolute poverty, they are still facing relatively high levels of instability. Hence, the tasks of preventing a return to poverty and alleviating relative poverty are paramount.

### 5.2. Spatio-Temporal Evolution Pattern under Different Development Goals

#### 5.2.1. Spatio-Temporal Distribution of Poverty-Stricken Households

**Figure 3:**
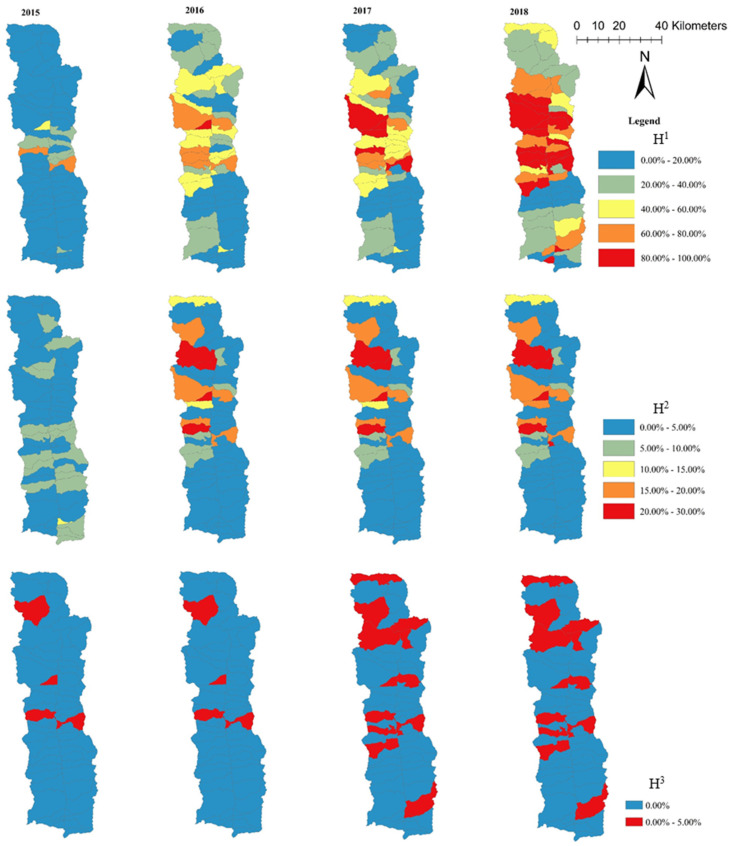
Spatial distribution of H^1^, H^2^, and H^3^ among households in different administrative villages from 2015 to 2018.

The spatio-temporal distribution of target realization rate H^1^, H^2^, and H^3^ of poverty-stricken households at the administrative village scale from 2015 to 2018 is shown in Figure 3. It can be inferred that the spatial distribution of the goal realization rate of poverty-stricken households with different development goals in different years was quite different. 

From the spatial distribution of H^1^, the target realization rate of most administrative villages in 2015 was lower than 20%. The target realization rate of the administrative villages in Shangpa Town was between 20–80%, i.e., obviously higher than that of other towns. In 2016, except for Jikedi, all areas had shown obvious improvement, although the higher administrative villages were still mainly distributed in Shangpa. In 2017, the target achievement rate in the southern and northern regions of the study area did not significantly improve, while that in the central region increased significantly, reaching more than 80% in some villages. Most of the villages with a target achievement rate of more than 90% in 2018 are in Shangpa and Lumadeng. The H1 rate of the short-term goals of 12 villages reached more than 90%, which was consistent with the result of changes in the country’s poverty index. A target rate of less than 20% was observed for the villages distributed to the south of Jiakedi. The realization rate was higher in the middle and lower in the south and north. According to the change characteristics of distribution density from 2015 to 2018, spatial differences in the process of eliminating absolute poverty in Fugong County increased in size during the period dedicated to achieving short-term goal T_1_.

From the spatial distribution of H^2^, the target realization rate in 2015 was consistently lower than 10% and could mainly be divided into two levels: 0–5% and 5–10%. In 2016, the villages with high target realization rates were mainly distributed in the north of the study area. In 2017, the target rate of some villages reached more than 20%, mainly in the north and central regions. The number of administrative villages that achieved a target rate of more than 20% in 2018 did not change significantly, and the overall level of development was still low. Generally, the distribution pattern from 2015 to 2018 was quite different, characterized by the spatial distribution of “high in the middle and North-low in the South”. Maji and Shangpa gradually formed a new growth pole under the T_2_ goal.

According to the spatial distribution of H^3^, from 2015 to 2018, the T_3_ long-term goal realization rate households of in most villages was 0. The spatial distribution of the administrative villages for poor households to achieve the long-term development goals was mainly segmented and sporadic, and the center of spatial distribution was found to be gradually shifting to the middle and north.

On the whole, farmer households with high realization rates under the T_1_ goal were mainly distributed in the central region of Fugong county. Due to the implementation of poverty alleviation policies, they showed a relatively significant spatial agglomeration distribution. Under the T_2_ and T_3_ development goals, such households were mainly distributed in the central and northern regions and showed a segmental and sporadic distribution.

#### 5.2.2. Spatio-Temporal Correlation of the Distribution of Poverty-Stricken Households 

Firstly, the global spatio-temporal dependence of each target realization rate was obtained based on a global spatial and temporal autocorrelation analysis. As shown in Table 3, the STI and Moran’s I values of H^1^ in 2015–2018 all passed a 1% confidence test. This indicated that the distribution of H^1^ presented a significantly strong spatial dependence, and the spatio-temporal distribution pattern was aggregated. With poverty reduction, spatial dependence under the T_1_development goal was found to be gradually increasing. 

STI of H^2^ and Moran’s I in 2017 passed the 5% confidence test. In 2018, Moran’s I passed the 1% confidence test but failed it in the other years. This indicates that there was no significant spatial dependence in 2015 to 2016, but that there was spatial dependence in the distribution of H^2^ from 2017 to 2018. Although the distribution pattern of H^2^ was also aggregated, the spatial dependence was lower than that of H^1^. Similarly, the spatial dependence of such households under the T_2_ development goal was shown to be gradually increasing.

As far as STI of H^3^ and Moran’s I in 2015~2018 are concerned, they all failed the significance test and presented random distribution. This indicates that there were no significant spatial dependence or spatial aggregation.

**Figure 4:**
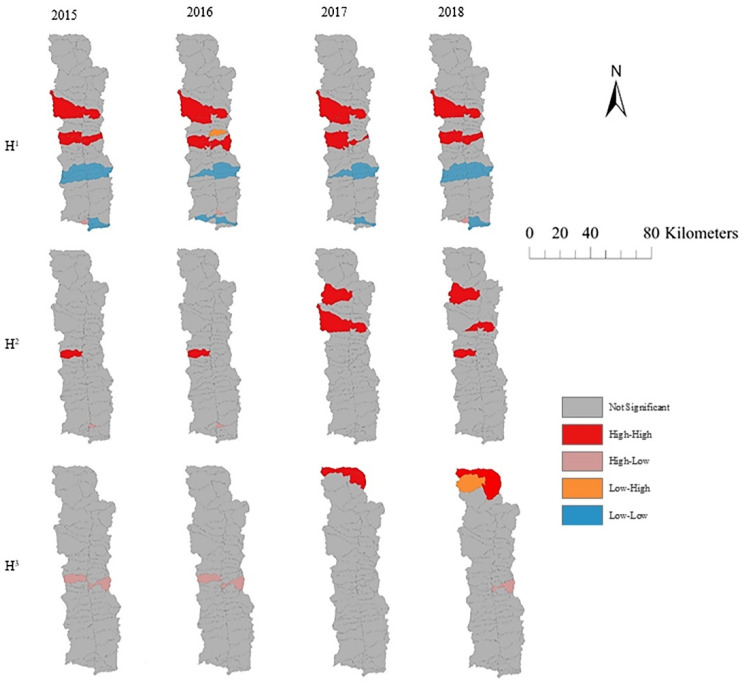
Local spatio-temporal autocorrelation distribution of H^1^, H^2^, and H^3^.

The local spatio-temporal autocorrelation statuses of H^1^, H^2^, and H^3^ were obtained by the local autocorrelation method, as shown in Figure 4. The local autocorrelation distribution types of H^1^ from 2015 to 2018 were mainly of the high–high or low–low types; the distribution of the former was concentrated in Shangpa and Lumadeng, while the latter mostly appeared in Jikedi. From the perspective of individuals, the majority of farmers in Shangpa and Lumadeng achieved the goal of eliminating absolute poverty during the study period, but the factors that made this possible were different. The driving force for farmers to escape absolute poverty in Shangpa Town was mainly the improvement of housing safety in the region but was also partly due to the construction of sanitary toilets and the improvement of family health conditions. Additionally, household income in Lumadeng improved significantly, as did housing safety, family health, and the education level of the labor force. As the main area of low–low-type local autocorrelation distribution, the absolute-poverty households in Jikedi were mainly characterized by poor family health, a lack of housing security, and income insecurity.

With increased poverty reduction and development capacity among poor farmers, the high–high types gradually increased, as did their spatial dependence. The local autocorrelation distribution type of H^2^ was mainly high–high, presenting an aggregation distribution. From 2015 to 2016, there were few high–high types, and there was only one administrative village. After 2017, the high–high types gradually increased in number, showing significant spatial dependence and aggregation characteristics. H^3^ showed a random distribution pattern, while the local autocorrelation showed no significant spatial dependence. Specifically, a relatively stable high–high spatial region was formed in Shidi, while spatial hot spots were also formed in Lamadi, Watuwa and Chisadi (Lumadeng) from 2017 to 2018. From the perspective of individual farmers, the popularization of sanitary toilets in Shidi of Shangpa greatly contributed to poverty reduction among farmers in the mid-term development process. The formation of spatial hot spots in Lamadi, Watuwa, and Chisadi (Lumadeng) was mainly due to the significant increase in the incomes of some farmers and the construction of electric power projects. 

In general, under the T_1_development goals, villages with high target realization rates were mainly distributed in the central region of Fugong County and presented a significant spatial agglomeration distribution feature. The high–high type was concentrated in Shangpa and Lumadeng, while the low–low type mostly appeared in Jikedi. In the development process of the T_2_ and T_3_ goals, the realization rates of villages were generally low. Under the mid-term development goals, villages with target realization rates of more than 20% were mainly distributed in the north and central regions, and hot spots existed in Lumadeng and Shangpa. Under the T_3_ goal, villages with high realization rates showed segmental and sporadic distribution characteristics and did not show significant spatial dependence.

### 5.3. Poverty Causing Factors Analysis

In order to identify the main poverty causing factors and accurately determine poverty types in Fugong County, the development obstacle degree (i.e., *C_j_*^1^, *C_j_*^2^, *C_j_*^3^) and target realization rate (i.e., *H*^1^*_j_*, *H*^2^*_j_*, *H*^3^*_j_*) of each indicator *j* under the standards of *T*_1_, *T*_2_ and *T*_3_ were calculated using Formula (13); the results are shown in Table 4. Sorting the results from large to small under the *T*_1_ target, it may be seen that the main impoverishment factors were *X*_5_, *X*_4_, *X*_1_, *X*_8_ and *X*_7_, while the main impoverishment factors under the *T*_2_ target were *X*_5_, X_4,_ *X*_8_, *X*_1_, *X*_7_, *X*_3_, and *X*_8_, *X_5_*, *X*_4_, *X*_1_, *X*_7_ and *X*_3_ under the *T*_3_ target.

On the whole, the main factors causing poverty in Fugong County are per capita net income (*X_8_*), safe housing (*X_1_*), sanitary toilets (*X_4_*), and average years of education of the labor force (*X*_5_). With regional poverty reduction and development initiatives, the obstacles to decreasing poverty in terms of housing structure, per capita net income, access to electricity and drinking water have been gradually overcome, while years of education of labor force, family health, and sanitary toilets are gradually increasing. Under different development goals, the main factors causing poverty in the region are diverse; notably, net income per capita showed significant variation. 

Below, some typical poverty causing factors are analyzed.

(1) *X*_5_ (Years of education of labor force). In Fugong County, the average duration of education has reached the six-year poverty alleviation standard in just 13.68% of poverty-stricken households, and the realization rate of medium- and long-term goals is also relatively low. Compared with the average level of rural residents in Yunnan Province, there is a large gap. From the perspective of the development contribution of indicators, the obstacles to poverty increased gradually from 2015 to 2018. In 2018, *C*^1^, *C*^2^, and *C*^3^, of *X*_5_ were 32.66%, 27.99%, and 24.56%, respectively, which indicated that the education level of the labor force was a main factor limiting the development of the poor households in Fugong County.

(2) *X*_4_ (Sanitary toilets). The proportion of households with sanitary toilets was 21.79 % in Fugong County, 21.3% in Yunnan Province, and 31.7 % in rural areas nationwide. Across the country, the penetration rate of sanitary toilets is relatively low. Therefore, sanitary toilets are not only a limiting factor for development of Fugong County, but also an obstacle to the development of rural areas throughout the country. From 2015 to 2018, the obstacle degree of *X*_4_ to poverty gradually increased, and was also at a high level in 2018, which indicated the significant impact of the indicator on the degree of poverty among farmers.

(3) *X*_8_ (Per capita village income). The average per capita net income among poor households in Fugong County was 4166 RMB yuan, with 64.23% of households exceeding the absolute poverty line in 2018 (3533 RMB yuan), 24.23% of households reaching the national average level for rural residents, and 0.96% reaching the average level for rural residents in Yunnan Province. Overall, the income level among poor households in Fugong County is far lower than the national average level for rural areas, and there is a large gap with the provincial average level. Under the short-term development goals, the obstacles to poverty have been gradually overcome from 2015 to 2018. Economic obstacles in 2018 were lower than *X*_6_, *X*_5_, *X*_1_, i.e., they were not the most significant factors contributing to poverty. However, under the medium- and long-term goals, economic income is particularly significant. There is therefore an urgent need to sustainably increase the incomes of poor farmers.

(4) *X*_1_ (Building structure). Through the removal of dilapidated houses and other projects, the housing conditions among impoverished households in Fugong County have greatly improved, but there are still many households for whom housing safety is not guaranteed. From the perspective of spatial distribution, the distribution pattern of safe housing development is consistent with that of comprehensive development, i.e., high in the middle section and low in the north and south sections; this is also affected by location conditions.

(5) *X*_7_ (Family Health). From 2015 to 2018, the degree of poverty obstacles increased. In 2018, the H^1^*_j_* of *X*_7_ was 74.29%, and 25.71% of poor families had family members suffering from a serious illness or disability. Poverty caused by illness or disability is still relatively widespread in the region. From the perspective of spatial distribution, the contribution of the southern section was significantly higher than that of the middle and the northern sections, and families with severe diseases or disabilities were shown to be concentrated in Shiyue, in the northern section.

## 6. Discussions

### 6.1. The Advantages and Disadvantages of G-TOPSIS Model

In this paper, the G-TOPSIS model was constructed to identify the degree and causes of poverty among farmers under the different stages of targets. Combined with a spatio-temporal correlation and obstacle degree analysis, the model was shown to be effective at measuring the poverty alleviation development process and identifying spatio-temporal evolution trends among multidimensional poverty-stricken farmers. Several former studies have shown that TOPSIS can effectively reflect the gap between the current situation and the goal, making it widely applicable in various areas [5,9,30]. However, the use of Euclidean distance in traditional TOPSIS often obscures the overall trends in data due to the huge deviation of certain indexes [10,23]. By comparison, G-TOPSIS can more objectively reflect the deprivation of each index from the object and is more aligned with the complex poverty characteristics of rural farmers in China. The obtained results confirmed our hypothesis. When applied to multidimensional analyses in multiobjective scenarios, the method is capable of answering the questions “what are the characteristics of poverty, how does it evolve over time and space, and what obstacles exist for its reduction”. Compared with research using only poverty measurement methods [3,5,6,31] or spatial analyses [8,29], our method can effectively reveal poverty reduction spatiotemporal patterns and influencing factors at different objective stages; this is crucial for practical policy making. 

It should be noted that a few factors in the multidimensional poverty index system used in this paper were not fully analyzed limited due to the limited availability of data. As such, the influence paths of poverty causing factors were not fully examined. Further research should focus on analyzing the evolutionary paths at different zoning scales. 

### 6.2. Policy Implications

Based on a multiobjective spatio-temporal evolution analysis method, this paper examined poverty reduction among poverty-stricken farmer households under different development goals in Fugong County in Yunnan Province, China. The results showed that the region solved its overall absolute poverty problem from 2015 to 2018, forming a regional growth hotspot according to its resources and industrial structure and accelerating the rate of progress in terms of target achievement in the surrounding areas. However, there are still some problems that need to be addressed if we are to establish a long-term mechanism to overcome relative poverty. 

At a town scale, during the process of eliminating absolute poverty, the spatial and temporal agglomeration characteristics were relatively significant, on the whole. Some towns, due to the infrastructure and public service system in the region [32,33,34], have certain industrial advantages and achieved a higher proportion of absolute poverty elimination. However, the fragile ecosystem and frequent natural disasters make it difficult for absolutely poverty-stricken households to benefit from an industrial economy based on on local resources. Comprehensive and reasonable evaluations and analyses of regional ecological environments are key to eliminating absolute poverty [22,31,35,36]. On the one hand, relocations can bring about more widespread safe living and production environments [36,37]; on the other hand, by increasing the extent of ecological protection regulations and restoration jobs, poor people living in key ecological function zones who can work can be lifted out of poverty by means of transfer payments [38].

At a village scale, during the process of the development for medium and long-term goals, the spatial agglomeration features were relatively weak. Some villages have effectively alleviated the relative poverty of some farmers by implementing industrial poverty alleviation measures; however, industry selection fails to combine the regional industrial resource base and the existing industrial structure to form a sustainable development industry [39]. Moreover, it fails to carry out vocational education in combination with existing industries, which is the key to the mutual promotion of human resources and industrial development [40,41].

At the individual level, the farmers seeking to overcome absolute poverty generally experience poor conditions, such as low education levels and a lack of stable income [42,43]. Some individuals are also poor due to factors such as housing structure and family health. Poverty resulting from old age, disease, study, and disability are commonplace in absolute poverty-stricken households [44,45]. Under the medium and long-term goals, with the improvement of living conditions and family health, the gap between actual incomes and the goal is the main cause of poverty among peasant households. However, relatively poverty-stricken households persist due to factors such as housing safety, family health, a lack of sanitary toilets, and other diversified personality characteristics [33,46,47,48,49]. 

Therefore, to eliminate absolute poverty, reasonable planning and construction of infrastructure and industrial development based on the characteristics of the regional natural environment are key. However, for regions lacking the conditions required to overcome poverty, relocation or adding ecological protection posts should be considered, according to local conditions. On the other hand, for regions that have overcome overall absolute poverty, the identification and formulation of policies to address absolute poverty should be promoted by individuals based on the regional characteristics. In the process of getting rid of relative poverty and realizing rural revitalization, the key to achieving the high-quality and sustainable development of a rural economy is to select industries according to the industrial structure of the macroeconomy and to combine them with vocational education.

## 7. Conclusions 

In this paper, we built a G-TOPSIS multiobjective poverty reduction measurement model to examine and analyze the poverty reduction effect on poverty-stricken farmers in the study area under different goal stages. Meanwhile, we introduced spatio-temporal correlation analysis model to reveal the spatial and temporal agglomeration characteristics of multidimensional poverty among farmers under different development goals. Then, the obstacle degree model was utilized to comprehensively detect and analyze poverty causing factors under the different goal stages. Our case test of Fugong County yielded the following results: (1) Although great progress was made in poverty alleviation during the study period, some rural households remain in absolute poverty. Hence, total eradication of absolute poverty is the most urgent goal of poverty reduction. Moreover, some rural households who have been lifted out of poverty are still experiencing relative poverty, as referenced to the national level, with high vulnerability. (2) Farmers with higher realization rates are mainly distributed in central and northern regions. Under the short- and medium-term development goals, Shangpa and Lumadeng showed a high-agglomeration trend, while the low-agglomeration type mostly appeared in Jikedi, showing a random distribution under the high development goals. (3) The main poverty causing factors under the short-term development goals are per capita net income, safe housing, sanitary toilets, years of education of labor force, and family health. Under the mid- and long-term goals, per capita net income, labor force education, and safe housing are the major development limitations of Fugong County. These factors in the county remain far below the national average levels. These findings have the potential to provide theoretical foundations for practical policy making for national poverty reduction.

## Figures and Tables

**Table 1:** Multiobjective poverty reduction measurement indicator system.

Dimension	Indicators	Indicator Interpretation and Assignment	Combination Weight	*T*_1_: Short-Term Goals	*T*_2_: Medium Term Goal	*T*_3_: Long-Term Goals
Housing safety	*X_1_*—Building structure	Grade A = 1 Grade B = 0.75, Grade C = 0.5 Grade D = 0.25 C and D comprised “dangerous” houses;The values were assigned to the four grades, i.e., A, B, C and D, as designated by the appraisal standards for “dangerous” houses. These standards were established based on whether the bearing capacity of the building structure could meet the requirements for daily use, as issued by the Ministry of Housing and Urban-Rural Development of the People’s Republic of China.	0.125	0.75	1	1
Living condition	*X_2_*—Drinking water safety	Safe = 1, Not Safe = 0; Drinking water safety means adequate and timely access to drinking water without long-term effects on human health	0.200	100%	100%	100%
	*X_3_*—Electricity supply	Both for production and daily use = 1, Only for daily use = 0.5, No electricity = 0; The power consumption for production is 380 V, and the power consumption for living is 220 V	0.075	0.5	0.5	1
	*X_4_*—Toilet	No Toilet = 0, Available Toilet = 1 Toilets include flush and dry toilets	0.050	1	1	1
Education	*X_5_*—Average Years of Education of the Labor Force (Years)	The average of the total number of years of academic education in the labor force	0.100	6	7.19	7.6
	*X_6_*—The Enrolment rate of compulsory education (%)	No dropout from compulsory education of school-aged children = 1, dropout from compulsory education because of poverty = 0	0.075	1	1	1
Health condition	*X_7_*—Family health	Health = 1, Family members have chronic diseases = 0.5, Family members have disabilities = 0.25, A family member is seriously ill = 0	0.100	1	1	1
Family income	*X_8_*—Per Capita Net Income (Yuan)	The average income of the family members in the current year	0.175	2855/	7070/	10772/
				3000/	7874/	12363/
				3335/	8695/	13432/
				3533/	9862	14600/
Social Security	*X_9_*—Participation rate of rural cooperative medical insurance (%)	Percentage of family members participating in the new rural cooperative medical care system or, for urban residents, the percentage of those with medical insurance	0.050	100%	100%	100%
	*X_10_*—Participation rate of old-age insurance for family members (%)	Percentage of family members with rural old-age insurance or urban old-age insurance	0.050	100%	100%	100%

Note: Indicator grading values refer to the China Statistical Yearbook, China Statistical Yearbook of Poverty Allowance and Development, Yunnan Statistical Yearbook, identification standard of dangerous houses, evaluation standard of rural drinking water safety, rural electrification standard, rural household toilet hygiene standard, and other national industry standards.

**Table 2:** Development levels among poor households in Fugong County from 2015 to 2018.

Development Level	2015	2016	2017	2018	Definition
high	0.04%	0.12%	0.20%	0.24%	higher than the national average level
relatively high	0.06%	1.45%	3.33%	6.99%	higher than the provincial average level but lower than the national average level
relatively low	6.99%	23.60%	27.54%	47.60%	higher than the poverty alleviation standard but lower than the average level of Yunnan Province
low	92.00%	74.83%	68.93%	45.17%	Below the national poverty alleviation standard

**Table 3:** Moran’s I statistics.

	STI	Moran’s I in 2015	Moran’s I in 2015	Moran’s I in 2017	Moran’s I in 2018
H^1^	0.434 **	0.335 **	0.430 **	0.486 **	0.581 **
Z	5.578	4.149	5.165	5.774	6.894
H^2^	0.126 *	0.027	0.125	0.176 *	0.223 **
Z	1.987	0.577	1.932	2.252	2.827
H^3^	0.067	−0.064	0.071	0.064	−0.062
Z	0.699	−0.692	1.443	1.019	−0.581

Note: ** means passing the significance test at the 1% level and * means passing the significance test at the 5% level.

**Table 4:** Contribution degree and target realization rate of each indicator from 2015 to 2018.

	Indcicator (%)	*X_1_* (Building Structure)	*X_2_* (Drinking Water Safety)	*X_3_* (Electricity Supply)	*X_4_* (Toilet)	*X_5_* (Average Years of Education of the Labor Force)	*X_6_* (Enrolment Rate of Compulsory Education)	*X_7_* (Family Health)	*X_8_* (Per Capita Net Income)	*X_9_* (Participation Rate of Rural Cooperative Medical Insurance)	*X_10_* (Participation Rate of Old-Age Insurance for Family Members)
Year											
2015	*C_j_^1^*	22.68	4.58	0.04	10.62	21.02	0.00	6.76	34.31	0.00	0.00
	*C_j_^2^*	20.13	4.06	2.66	9.43	20.22	0.00	6.00	37.50	0.00	0.00
	*C_j_^3^*	24.25	3.84	2.51	8.90	19.31	0.00	5.66	35.53	0.00	0.00
	H^1^*_j_*	25.26	90.57	99.79	12.49	13.42	100.00	72.16	19.22	100.00	100.00
	H^2^*_j_*	25.26	90.57	83.53	12.49	6.17	100.00	72.16	0.57	100.00	100.00
	H^3^*_j_*	4.58	90.57	83.53	12.49	5.02	100.00	72.16	0.16	100.00	100.00
2016	*C_j_^1^*	23.06	0.62	0.00	12.79	27.57	0.00	6.39	29.58	0.00	0.00
	*C_j_^2^*	18.67	0.50	0.14	10.35	23.63	0.00	5.17	41.54	0.00	0.00
	*C_j_^3^*	26.16	0.45	0.13	9.23	21.32	0.00	4.61	38.12	0.00	0.00
	H^1^*_j_*	39.44	98.98	99.99	16.05	9.51	100.00	79.03	44.52	100.00	100.00
	H^2^*_j_*	39.44	98.98	99.24	16.05	4.17	100.00	79.03	3.73	100.00	100.00
	H^3^*_j_*	4.79	98.98	99.24	16.05	3.02	100.00	79.03	0.91	100.00	100.00
2017	*C_j_^1^*	21.28	0.48	0.01	12.90	27.17	0.00	8.17	29.99	0.00	0.00
	*C_j_^2^*	17.27	0.39	0.24	10.48	23.79	0.00	6.63	41.20	0.00	0.00
	*C_j_^3^*	25.88	0.34	0.21	9.16	21.05	0.00	5.80	37.57	0.00	0.00
	H^1^*_j_*	44.92	99.23	99.97	16.50	12.09	100.00	73.57	44.56	100.00	100.00
	H^2^*_j_*	44.92	99.23	98.72	16.50	5.17	100.00	73.57	6.17	100.00	100.00
	H^3^*_j_*	5.60	99.23	98.72	16.50	4.02	100.00	73.57	2.11	100.00	100.00
2018	*C_j_^1^*	18.28	0.00	0.00	14.95	33.00	0.00	9.83	23.93	0.00	0.00
	*C_j_^2^*	13.41	0.00	4.92	10.97	26.31	0.00	7.21	37.18	0.00	0.00
	*C_j_^3^*	24.98	0.00	3.76	8.37	20.32	0.00	5.50	37.08	0.00	0.00
	H^1^*_j_*	61.75	100.00	100.00	21.79	13.68	100.00	74.29	64.23	100.00	100.00
	H^2^*_j_*	61.75	100.00	76.59	21.79	6.17	100.00	74.29	24.23	100.00	100.00
	H^3^*_j_*	6.60	100.00	76.59	21.79	5.02	100.00	74.29	0.96	100.00	100.00
